# Editor's Note on ‘Mechanisms of transcriptional repression of cell-cycle G_2_/M promoters by p63’

**DOI:** 10.1093/nar/gkad542

**Published:** 2023-07-10

*Nucleic Acids Research*, Volume 34, Issue 3, 1 February 2006, Pages 928–938, https://doi.org/10.1093/nar/gkj477

The Editors were alerted in July 2022 that panel ΔNp63α of Figure 4A and sub-panel YC in panel NF-YB/C D112N of Figure 4C appear to be identical.

The Editors subsequently investigated and liaised with the authors who replied promptly. The authors acknowledged with regret errors in the preparation of Figures 4A and 4C.

Figure 4A: Below is a picture of the lab book with the western blots of the immunoprecipitation experiments shown in Figure 4A, with the films of YA (Left), p63 (Central) and YB (Right) original blots. 4A4 is the anti-p63 antibody. The 5 lanes are -left to right- Load (L), Flow Through (FT), Bound (B), Flow Through (FT), Bound (B).

This image confirms the validity of the results presented in Figure 4A.


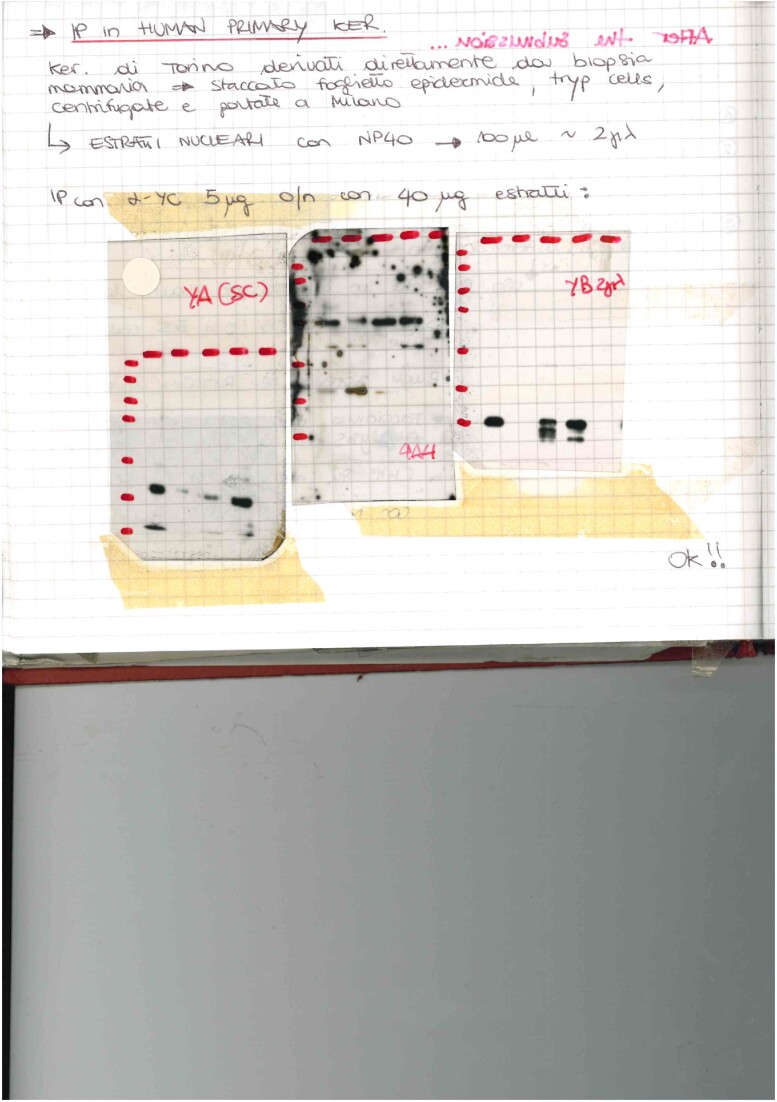


Figure 4C: Below is the raw data of the D112N panel which should have been used for Figure 4C. The upper panel shows the data of p63 (4A4). The 5 lanes are -left to right- Load (L), Flow Through (FT), Bound (B), Flow Through (FT), Bound (B) as above. In the lower panel, the blot was probed with the internal control YB, stripped, and then probed with YC antibodies. Stripping did not eliminate the strong YB signal. The 32 kD band of YB and the smaller 20/22 kD band of the NF-YC mutant (red asterisk), both recombinant proteins used for the IP, are shown.

The p63 and YB panels of Figure 4C are genuine but the wrong panel was erroneously inserted for YC.


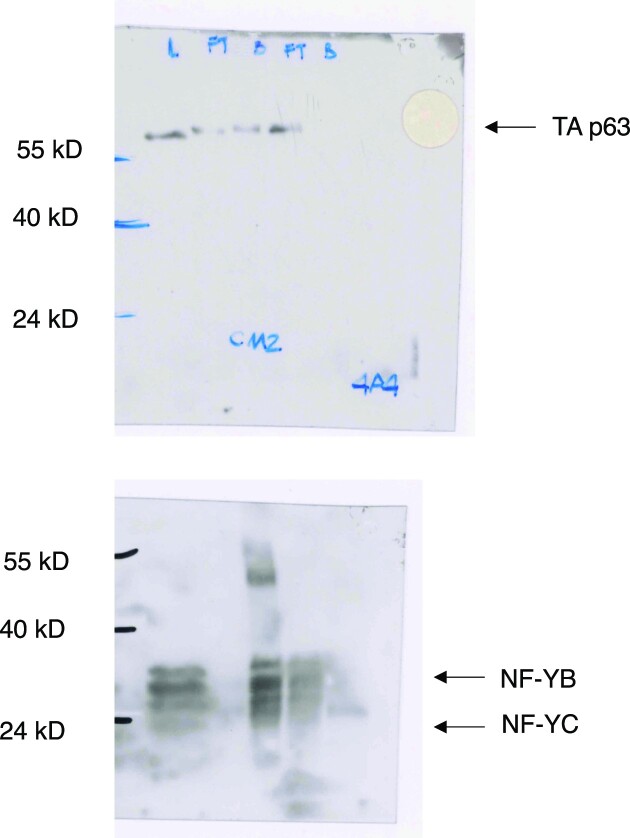



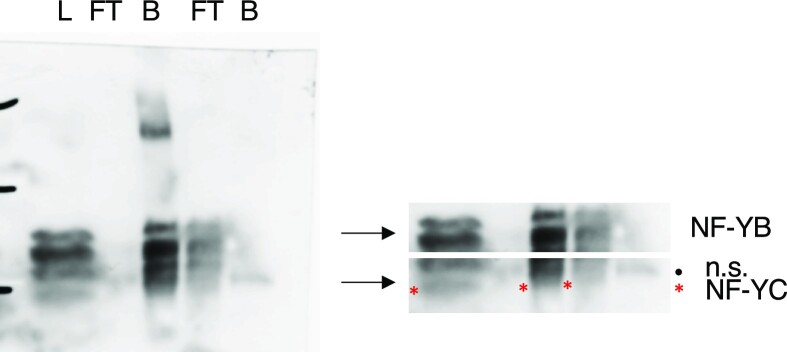


The Editors conclude that the quality of the replacement image for Figure 4C is not sufficient to confirm the results of this specific experiment. While these issues may not affect the results or conclusion of the study as a whole, the Editors advise readers to examine Figure 4C with care.

The Editors were also alerted to the problems listed below but did not find evidence to support the allegations.

Figure 3E: NF-YB panel, the bands in lanes 4 and 5 appear to be similar.
Figure 4C:
  - The band in lane 3 of the NF-YB/C5 TAp63alpha panel appears to be similar to the band in lane 2 of NF-YB/C D112N Tap63alpha panel.
  - The band in lane 4 of the NF-YB/C5 TAp63alpha panel appears to be similar to the band in lane 2 of the NF-YB/C D112N Tap63alpha panel.
Figure 6: the panels appear computer-generated.
Figure 7: the panels appear computer-generated.

Concerns about Figures 1 and 2 were addressed in an earlier correction notice: https://doi.org/10.1093/nar/gkt991.

Julian E. Sale and Barry L. Stoddard

Senior Executive Editors

